# CXCL12/CXCR4 Axis Regulates Aggrecanase Activation and Cartilage Degradation in a Post-Traumatic Osteoarthritis Rat Model

**DOI:** 10.3390/ijms17101522

**Published:** 2016-09-29

**Authors:** Weiwei Lu, Jia Shi, Jinming Zhang, Zhengtao Lv, Fengjing Guo, Hui Huang, Wentao Zhu, Anmin Chen

**Affiliations:** Department of Orthopedics, Tongji Hospital, Tongji Medical College, Huazhong University of Science and Technology, Wuhan 430030, China; luweiwei0906@gmail.com (W.L.); 1990sj1025@163.com (J.S.); zhangjinming1988@gmail.com (J.Z.); 630105736lzt@gmail.com (Z.L.); fjguo@tjh.tjmu.edu.cn (F.G.); 2007hh@gmail.com (H.H.)

**Keywords:** SDF-1α/CXCR4, post-traumatic osteoarthritis (PTOA), ADAMTS-4/5

## Abstract

We evaluated the role of the CXCL12/CXCR4 (C-X-C motif chemokine ligand 12/C-X-C chemokine receptor type 4) axis in aggrecanase-mediated cartilage degradation, and explored the underlying mechanism in a post-traumatic osteoarthritis rat model. Expression of CXCL12/CXCR4 and ADAMTS-5 was analyzed in the knees of osteoarthritic and non-arthritic rats using Western blot, ELISA, immunohistochemistry and immunofluorescence. Rodent studies were performed using Sprague-Dawley rats, with animals divided into three groups: Destabilization of the medial meniscus/AMD3100-treated (DMM/AMD3100-treated), DMM/PBS-treated, and sham controls. Rats were sacrificed after eight weeks, and samples were collected for histology and immunohistochemistry analyses. IL-1-pretreated primary chondrocytes were cultured with untreated control, CXCL12a, siNC + CXCL12a, or siRNA CXCR4 + CXCL12a, and analyzed for expression of relevant markers and cellular pathways. Higher levels of CXCL12 were detected in the knee fluid of osteoarthritic subjects, with strong staining for CXCR4 in chondrocytes and CXCL12 in synoviocytes together with enhanced expression of ADAMTS-5. DMM/AMD3100-treated rats showed a significantly reduced immunological response, with minimal evidence of pathology in both histological and immunohistochemical analyses. Treatment with CXCL12a increased the expression of ACAN, RUNX-2, and ADAMTS-4/5 in IL-1-pretreated primary chondrocytes, together with a decrease in the expression of SOX-9. Molecular analyses revealed strong induction of NF-κB activation, along with phosphorylation of MAPKs, and activation of canonical Wnt/β-catenin signaling. In conclusion, inhibition of SDF-1α/CXCR4 signaling axis was able to inhibit aggrecanase expression and lessen cartilage degeneration in post-traumatic osteoarthritis rats.

## 1. Introduction

Osteoarthritis (OA) is the most common form of arthritis and a major cause of pain and disability in older adults, afflicting over 15% of the Western elderly population [1]. Degradation of the articular cartilage is cardinal to OA pathogenesis, but other joint tissues are constantly affected. The temporal and mechanistic relationships of changes in the different tissue compartments, in particular between bone and cartilage, are subjects of debate [2]. Joint inflammation of varying severity is present in patients with OA [3]. Lesions in ligaments and menisci also develop at the same time as cartilage lesions [4].

Much of this cartilage destruction appears to be a result of extracellular matrix loss mediated by uncontrolled production of matrix-degrading enzymes [5]. The two major matrix components, type II collagen and aggrecan, are responsible for the vast majority of the mechanical properties associated with cartilage. Type II collagen is present as triple helical fibrils that provide cartilage with its structure and tensile strength, while multiple aggrecan monomers bind to hyaluronan and link proteins to form huge aggregates that fill in the interstices of the collagen network, providing compressibility and elasticity.

The loss of aggrecan is considered a critical event in early-stage OA, as the presence of aggrecan plays an important role in the maintenance of collagen fibrils [6]. Among known aggrecanases, a disintegrin and metalloproteinase with a thrombospondin motif 5 (ADAMTS-5) exhibits the most efficient aggrecanase activity, and therefore is considered the most likely candidate underlying the pathological cleavage of aggrecan in OA [7,8,9,10,11].

C-X-C motif chemokine ligand 12 (CXCL12), also known as stromal derived factor-1 (SDF-1), is a member of the chemoattractant cytokine superfamily. Within joint tissues, CXCL12 is primarily expressed by synovial fibroblasts, while the G-protein coupled receptor CXCR4 is expressed in chondrocytes [12]. Although the mechanism of release remains poorly understood, overproduction of CXCL12 has been associated with a range of inflammatory cytokines, including IL-1β, IL-17, HIF-1α, and TNF-α [13,14,15]. The CXCL12/CXCR4 axis plays a pivotal role in the injury and repair of cartilage by acting as a chemoattractant of cells involved in inflammation and stem cell migration [5,16,17,18,19,20,21].

In animal models of post-traumatic OA (PTOA), the expression CXCL12 and CXCR4 proteins is significantly upregulated in cartilage tissues, with similar increases in the synovial fluid of osteoarthritic knee joints [22,23]. In this study, we hypothesized that aggrecanase-associated cartilage degradation could be prevented by blocking the interaction between CXCL12 and its receptor CXCR4. In this study, we used AMD3100 (the specific antagonist of CXCL12/CXCR4 axis) (11.2 mg/kg·day) to block CXCL12/CXCR4 axis. To test the hypothesis, we first analyzed the level of CXCL12/CXCR4 and ADAMTS-5 in subjects with OA compared to non-arthritic control subjects. Then we induced OA in rats through surgical destabilization of the medial meniscus (DMM), and evaluated the effects of AMD3100 on the progression of OA and aggrecan metabolism. Third, we cultured rat primary chondrocytes with untreated control, CXCL12a, siNC + CXCL12a, or siRNA CXCR4 + CXCL12a and confirmed ACAN, SOX-9, RUNX-2, and ADAMTS-4/5 mRNA and protein levels in vitro. Finally, we explored the role of NF-κB, MAPK, and canonical Wnt/β-catenin signaling in CXCL12/CXCR4-mediated ADAMTS activation.

## 2. Results

### 2.1. High Expression of CXCL12/CXCR4 and ADAMTS-5 in Osteoarthritis (OA) Rats

**Figure 1:**
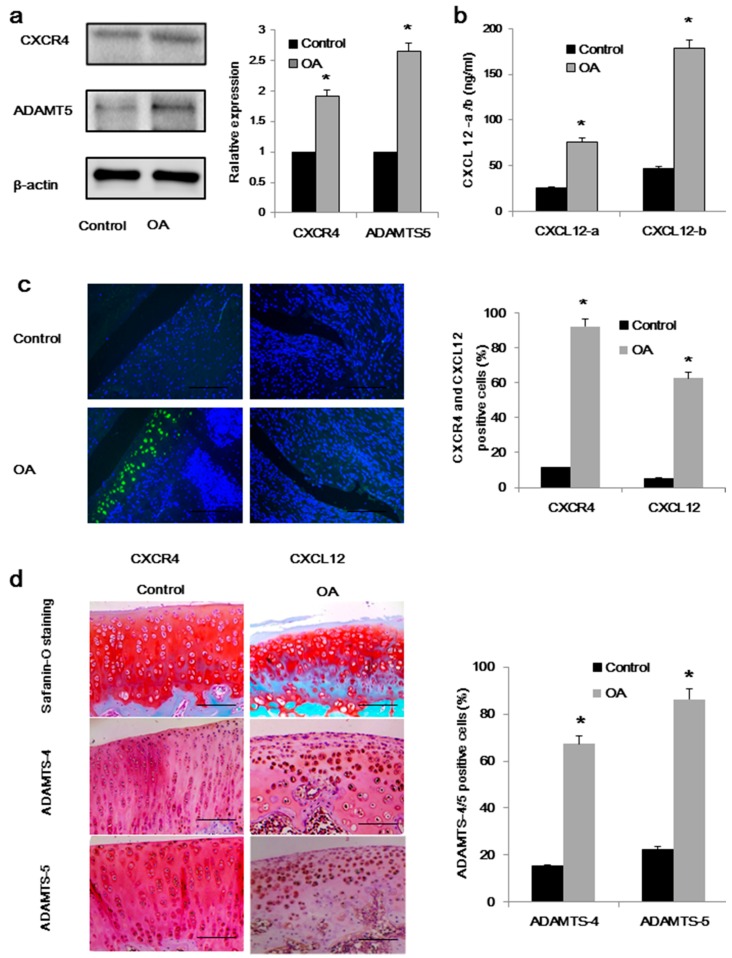
Expression of CXCL12/CXCR4 and ADAMTS-5 are upregulated in rat osteoarthritic samples: (**a**) Western blot analysis of ADAMTS-5, and CXCR4 expression in chondrocytes and synoviocytes isolated from osteoarthritis subjects and healthy control subjects, with quantification on the right. β-Actin served as a loading control (*n* = 4 for each group, * *p* < 0.05); (**b**) ELISA evaluated the CXCL12a/b levels from healthy control and osteoarthritic synovial fluid (*n* = 4 for each group, * *p* < 0.05); (**c**) immunofluorescence analysis of CXCL12-stained cells and CXCR4-stained cells in chondrocytes and synoviocytes representative of osteoarthritis and healthy control subjects, with quantification on the right (*n* = 5 for each group, * *p* < 0.05); and (**d**) staining for ADAMTS-4/5 in superficial chondrocytes of osteoarthritis and healthy control cartilage. The severity of osteoarthritis is moderate, as evaluated by the Mankin scores. Counterstaining is by safranin orange; scale bar, 100 μm. The number of ADAMTS-4/5-stained cells analyzed is provided on the right (*n* = 5 for each group, * *p* < 0.05).

We analyzed the expression of the CXCL12 and CXCR4 proteins in the synovial fluid from the knees of rats, along with the cartilage of OA rats and non-arthritic controls. Western blotting revealed significant increases in the expression of ADAMTS-5 and CXCR4 proteins in OA samples, relative to controls (Figure 1a), with elevated CXCL12 levels also detected in the synovial fluid of OA subjects (Figure 1b). The expressions of CXCL12 and CXCR4 in the synovial tissue and cartilage of OA rats and healthy controls were also studied by immunofluorescence. We detected strong cellular staining for CXCR4 in chondrocytes (92.2% of all chondrocytes), along with a significant increase in CXCL12 staining in synoviocytes (62.7% of all synoviocytes), relative to healthy controls (11.2% and 5.2% of all cartilage and synoviocytes, respectively; Figure 1c). Furthermore, we analyzed levels of ADAMTS-4/5 in a murine model of OA. In the superficial zone of the osteoarthritic cartilage of rats, we detected strong cellular staining for ADAMTS-4/5 (67.4% and 86.4% of the total chondrocytes) accompanied by heavy proteoglycan loss (Figure 1d). These changes are considered typical of OA, and are directly related to the number of chondrocytes expressing ADAMTS-5. By comparison, healthy control subjects were largely negative for ADAMTS-4/5 (15.2% and 22.5% of the total chondrocytes; Figure 1d).

### 2.2. Inhibition of the CXCL12/CXCR4 Axis Results in Reduced Cartilage Destruction and Alleviates OA Severity

**Figure 2:**
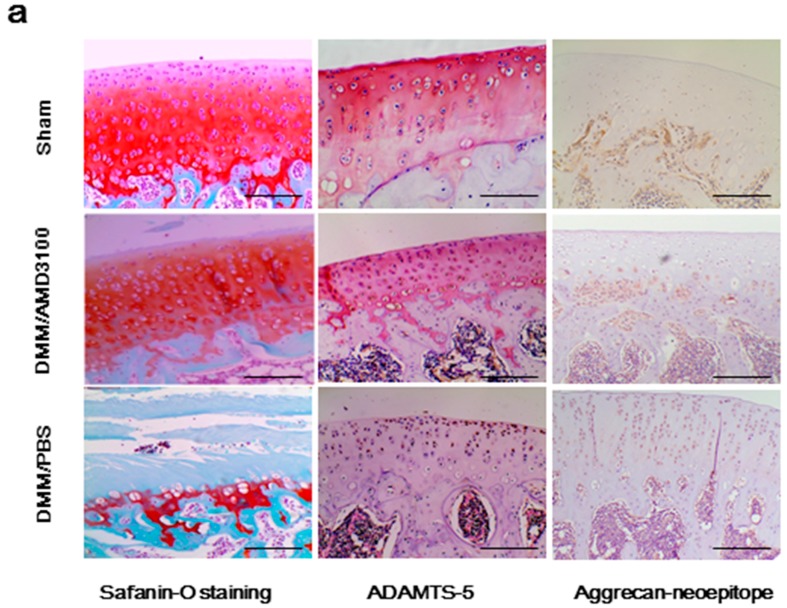

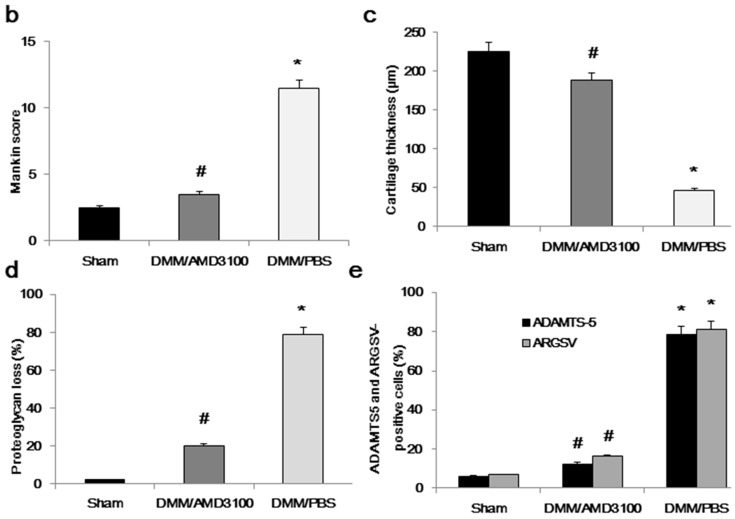
The blocking CXCL12/CXCR4 axis protects osteoarthritic cartilage from proteoglycan loss accompanied by reduced ADAMTS-5 and aggrecanneoepitope staining: (**a**) Safranin orange staining and Immunostaining of ADAMTS-5 and aggrecanneoepitope (374ARGSV) of knee sections in joints from sham, PBS-treated, and AMD3100-treated groups (scale bar, 100 μm); (**b**) Mankin score of articular cartilage in sham, PBS-treated, and AMD3100-treated groups (*n* = 9 for each group); (**c**) morphometric analysis of cartilage thickness in the three groups (*n* = 9 for each group); (**d**) percentage of proteoglycan loss in the knee cartilage of the three groups (*n* = 9 for each group); and (**e**) immunostainingof ADAMTS-5 and aggrecanneoepitope (374ARGSV) stained chondrocytes in the knee cartilage of sham, AMD3100-treated, and PBS-treated groups (*n* = 9 for each group). * DMM/PBS different from sham (*p* < 0.05); ^#^ DMM/3100 different from DMM/PBS (*p* < 0.05).

Safranin orange staining and immunohistochemistry revealed typical signs of proteoglycan loss and severe cartilage damage accompanied by enhanced ADAMTS-5 and aggrecan neoepitope (^374^ARGSV) expression in the affected knees of DMM/PBS-treated rats. These effects were significantly lower in the knee cartilage of both DMM/AMD3100-treated rats and sham controls (Figure 2a). Using the Mankin score, the degree of OA damage in each of these three groups was quantified by three blinded observers. Mankin scores were significantly lower (3.5 points; *p* < 0.05) in the DMM/AMD3100-treated group than in the DMM/PBS-treated rats (11.5 points); no significant differences were found between the DMM/AMD3100-treated group and sham controls (*p* > 0.05; Figure 2b). Samples collected from DMM/PBS-treated rats also exhibited a significant reduction in cartilage thickness (Figure 2c), along with a substantial loss of proteoglycans (Figure 2d). AMD3100 treatment was largely protective against OA-like changes, as reflected by the low Mankin scores in this group (Figure 2b), along with greater preservation of cartilage thickness (Figure 2c). Quantification of safranin orange-stained cartilage in affected rat knees revealed a 78.9% decrease in proteoglycan loss among DMM/AMD3100-treated rats compared to the DMM/PBS-treated group (Figure 2d). Furthermore, ADAMTS-5 expression in the affected knees was largely inhibited by AMD3100 (*p* < 0.05; Figure 2e).

Next, to confirm that AMD3100 protected rats from ADAMTS-mediated aggrecan loss, we examined aggrecanase activation and the appearance of ADAMTS-mediated aggrecan cleavage products (Figure 2a). Immunohistochemistry analyses revealed conspicuous neo-epitope staining throughout the arthritic cartilage of DMM/PBS-treated animals, with much milder staining seen in both the DMM/AMD3100-treated and sham groups. Morphometric analysis of the cartilage sections revealed positive aggrecan neo-epitope staining in 81.2% of chondrocytes in the DMM/PBS-treated group, compared to only 16.2% in the DMM/AMD3100-treated group and 6.8% in sham controls. Furthermore, ADAMTS-5 expression was positively correlated with aggrecan neo-epitope expression (Figure 2e).

### 2.3. CXCR4 Knockdown Inhibits IL-Induced Expression of ACAN, RUNX-2, and ADAMTS-4/5, and Enhances the Expression of SOX-9

To determine whether CXCL12a affects aggrecanase activation in vitro, we examined the effects of CXCL12a on markers associated with cartilage metabolism, including ACAN, Runt-related transcription factor 2 (RUNX2), ADAMTS-4/5, and SRY-type HMG box 9 (SOX-9). Rat primary chondrocytes pretreated with IL-1 (24 h, 10 ng/mL) were cultured with CXCL12a, siNC + CXCL12a, or siRNA CXCR4 + CXCL12a in vitro, and assessed for the expression of ACAN, RUNX-2, ADAMTS-4/5, and SOX-9 mRNA and protein levels at 24 and 72 h.

**Figure 3:**
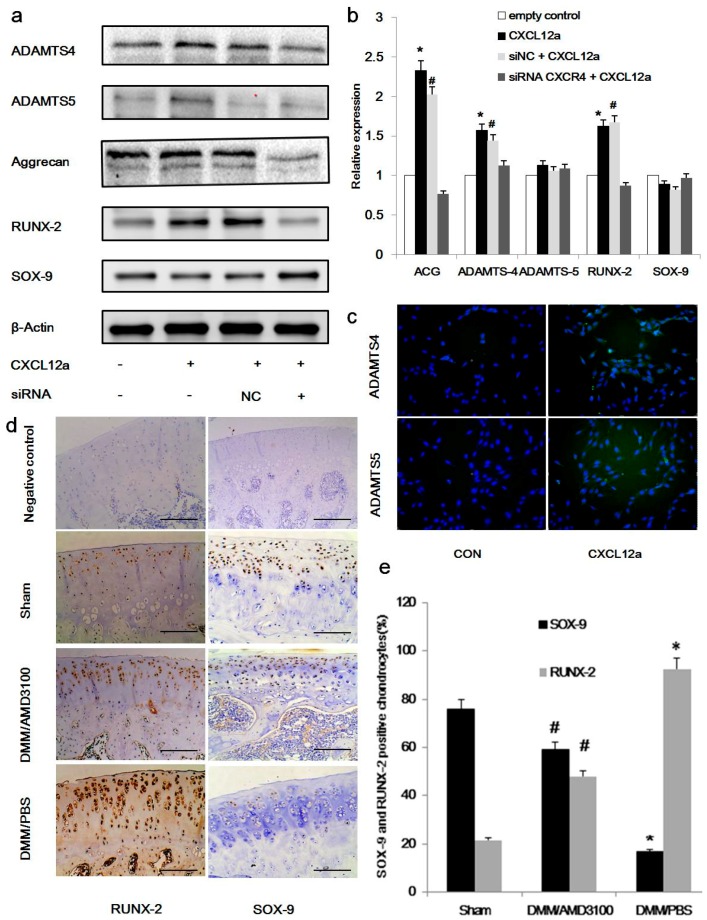
Blocking the CXCLL2/CXCR4 axis inhibits IL-induced expression of ACAN, RUNX-2, and ADAMTS-4/5, and enhances the expression of SOX-9: (**a**) Primary chondrocytes were cultured in the presence of empty control (−,−), CXCL12a (+,−), siNC + CXCL12a (+,NC), or siRNA CXCR4 + CXCL12a (+,+) for 72 h, and subjected to Western blot analysis to evaluate the protein level of ADAMTS-4/5, ACAN, SOX-9, and RUNX-2. β-Actin served as a loading control (*n* = 4 for each group); (**b**) Real-time PCR of ACAN, ADAMTS-4/5, SOX-9, and RUNX-2 in IL-1–induced primary cartilage treated with empty control, CXCL12a, siNC + CXCL12a, or siRNA CXCR4 + CXCL12a for 24 h; * CXCL12a different from empty control (*p* < 0.05); ^#^ siNC + CXCL12a different from siRNA CXCR4 + CXCL12a (*p* < 0.05); (**c**) Primary chondrocytes were cultured in the presence of the empty vector, or CXCL12a, for 48 h. Immunofluorescence staining was performed to study the expression of ADAMTS-4/5. Representative immunofluorescence images are shown. ADAMTS-4/5 was visualized with goat anti-rabbit IgG, and cell nuclei were stained blue by DAPI. Scale bar, 100 μm; (**d**) Cartilage sections from sham, AMD3100-treated, and PBS-treated groups were processed for immunohistochemical detection of the marker proteins RUNX-2 and SOX-9. Scale bar, 100 μm; and (**e**) Number of cells stained for RUNX-2 and SOX-9 in analyzed slices (*n* = 9 for each group, *p* < 0.05). * DMM/PBS different from sham (*p* < 0.05); ^#^ DMM/AMD3100 different from DMM /PBS (*p* < 0.05).

Treatment with 250 ng/mL CXCL12a significantly increased the expression of ACAN, RUNX-2, and ADAMTS-4 mRNA (*p* < 0.05); but it did not significantly affect SOX-9 and ADAMTS-5 expression. Chondrocytes treated with CXCR4 siRNA exhibited expression levels similar to that of the control group (Figure 3b). Regarding protein levels, primary chondrocytes cultured with 250 ng/mL CXCL12a for three days exhibited significant increases in the expression of ACAN, RUNX-2, and ADAMTS-4/5 (*p* < 0.05), combined with significant decreases in the expression on SOX-9 (Figure 3a, *p* < 0.05). Further analysis of primary chondrocytes pretreated with IL-1, followed by 250 ng/mL CXCL12a for three days, revealed strong induction of ADAMTS-4 and ADAMTS-5 by immunofluorescence. Taken together, these results indicate that the expressions of both ADAMTS-4 and ADAMTS-5 were enhanced by CXCL12a (Figure 3c). Immunohistochemical analyses of cartilage demonstrated that blocking the CXCL2/CXCR4 axis decreased the expression of Runx-2, while increased the expression of SOX-9 in rat PTOA model (Figure 3d,e).

### 2.4. CXCL12a-Mediated Aggrecanase Activation of Chondrocytes Is Dependent on the NF-κB, MAPK, and Wnt/β-Catenin Pathways

**Figure 4:**
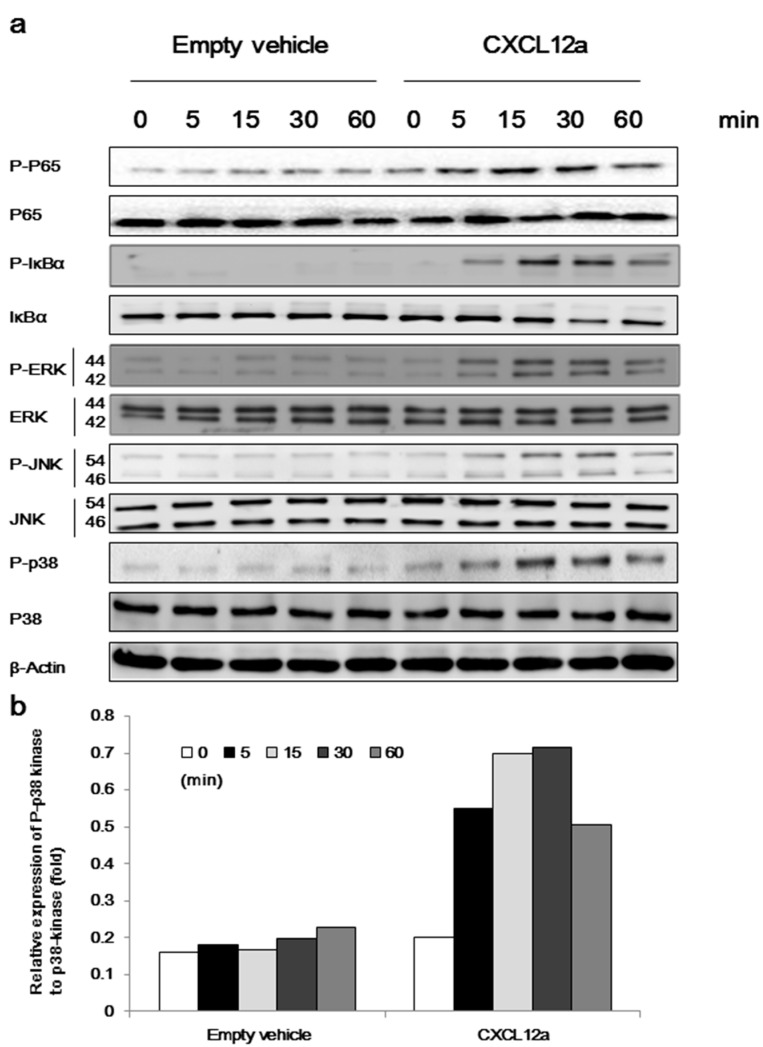
CXCL12a induces NF-κB activation and the phosphorylation of MAPKs. (**a**) CXCL12a induced phosphorylation of P65, IκBα, ERK, JNK, and p38. Pre-starved primary chondrocytes were treated with or without 250 ng/mL of CXCL12a for indicated times. The cell lysates were extracted for immunoblotting with the indicated antibodies and (**b**) Relative expression of P-p38 kinase to p38-kinase.

**Figure 5:**
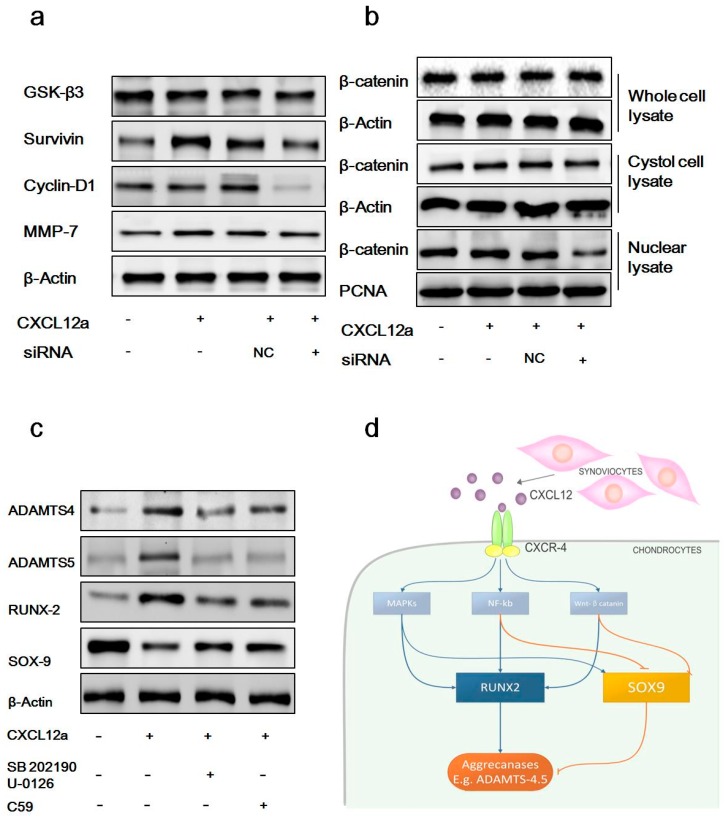
CXCL12a upregulates the activation of the Wnt/β-catenin pathway and blocking the MAPK or Wnt-catenin inhibits CXCL12a-induced expression of RUNX-2, and ADAMTS-4/5 and enhances the expression of SOX-9, and the model of CXCL12/CXCR4 axis-induced activation of aggrecanases. (**a**) Starved primary chondrocytes were exposed to CXCL12a (250 ng/mL), siRNA targeting CXCR4, CXCL12a + NC or CXCL12a + siRNA targeting CXCR4 for 48 h. The expression of molecules related to the Wnt/β-catenin pathway was demonstrated by the Western blotting assay; (**b**) The relative expression of β-catenin in different subcellular fractions was compared. β-Catenin and PCNA served as loading controls (*n* = 4 for each group); (**c**) Primary chondrocytes were cultured in the presence of empty control, CXCL12a, SB202190/U-0126 + CXCL12a, or C59 + CXCL12a for 72 h, and subjected to Western blot analyses to evaluate the protein levels of ADAMTS-4/5, ACAN, SOX-9, and RUNX-2. β-catenin served as a loading control (*n* = 4 for each group); and (**d**) CXCL12a produced in synovial fibroblasts binds to CXCR4 expressed by chondrocytes and subsequently activates the Wnt/β-catenin pathway, and leads to NF-κB activation and the phosphorylation of MAPKs, which are partly responsible for the increased expression of RUNX-2 and the downregulation of SOX-9. These changes ultimately promote the expression of aggrecanases.

Activation of the NF-κB, MAPK, and Wnt/β-catenin pathways plays a vital role in aggrecanase-mediated OA [24,25,26]. To elucidate the signaling pathway downstream of CXCL12a, we examined the activation of these pathways following treatment with CXCL12a. Primary chondrocytes were cultured in serum-free medium for 12 h, followed by the addition of 250 ng/mL CXCL12a for 48 h. This resulted in strong induction of p65 and IκBα phosphorylation, demonstrating that CXCL12a induces NF-κB activation (Figure 4a). Next, we examined the phosphorylation of MAPKs (ERK, JNK, and p38) in primary chondrocytes by Western blot. As shown in Figure 4b, CXCL12a induced the phosphorylation of ERK, JNK, and p38. These results appear to be similar to those of Kim et al. [24]. In addition to these pathways, CXCL12a was also found to activate the Wnt/β-catenin canonical pathway, including the activation of survivin, cyclin D1, and MMP-7 (*p* < 0.05 vs. empty-treated cells), while GSK-3β was slightly inhibited. In contrast, CXCR4 knockdown cells did not respond to CXCL12a stimulation (Figure 5a).

Then, we examined β-catenin expression in different subcellular fractions. Following treatment with 250 ng/mL CXCL12 for 48 h, nuclear and cytoplasmic proteins from primary chondrocytes were collected using NE-PER nuclear and cytoplasmic extraction reagents. Significant changes in β-catenin expression were observed in nuclei. No significant changes were seen in whole cell or cytoplasmic lysates compared to controls (Figure 5b).

Finally, we used MAPK and β-catenin inhibitors to demonstrate that MAPK and β-catenin induced by CXCL12a/CXCR4 axis could lead to SOX-9, RUNX-2, and ADAMTS-4/5 expression in the model. Rat primary chondrocytes pretreated with IL-1 (24 h, 10 ng/mL) were cultured with CXCL12a, SB202190/U-0126 + CXCL12a, or C59 + CXCL12a for 72 h in vitro, and assessed for the expression of SOX-9, RUNX-2, and ADAMTS-4/5 protein levels at 72 h. Primary chondrocytes cultured with 250 ng/mL CXCL12a for three days exhibited significant increases in the expression of RUNX-2, and ADAMTS-4/5, combined with significant decreases in the expression on SOX-9, while SB202190/U-0126 and C59 inhibited CXCL12a-induced expression of RUNX-2 and ADAMTS-4/5, and enhanced the expression of SOX-9.

## 3. Discussion

While collagen degradation in OA has so far proven irreversible, some aggrecan loss can be restored. This loss of aggrecan is considered a critical event in early-stage OA, as the presence of aggrecan plays an important role in the maintenance of collagen fibrils [3]. Therefore, maintaining the integrity of aggrecan is critical, not only as a means of attenuating disease severity but also for delaying or reducing the incidence of OA. In the present study, we first investigated the expression of CXCL12/CXCR4 and ADAMTS-5 in the synovial fluid of OA rats, as well as cartilage samples collected from both OA rats and non-arthritic controls. We found that the expressions of both CXCL12 and CXCR4 were increased in the context of OA, accompanied by stronger expression of ADAMTS-5, implying a strong correlation between the CXCL12/CXCR4 axis and aggrecanase-activated cartilage loss (Figure 1).

Based on these results, it was unclear whether CXCL12 and CXCR4 expression was simply correlated with aggrecanase activation in OA or was indeed a causative agent of disease pathology. To address this issue, we performed in vivo analyses, and found evidence that aggrecanase activation and cartilage loss are directly related to the CXCL12/CXCR4 axis. Blocking the CXCL12/CXCR4 axis attenuated these effects by inhibiting the degradation of aggrecan, further establishing the function of the CXCL12/CXCR4 pathway in PTOA progression (Figure 2). In a murine model of OA, less severe damage was observed in the DMM/AMD3100-treated group than in DMM/PBS-treated controls, indicating that AMD3100 effectively prevented PTOA-associated articular cartilage damage (Figure 2a). These results are consistent with earlier studies that showed that increased CXCL12/CXCR4 expression leads to reduced MMP expression and reduced matrix degradation [12,27], while inhibiting cartilage pathogenesis attenuated by the CXCL12/CXCR4 axis, increasing the overall severity of OA [18,28]. This is the first study that has focused on the role of the CXCL12/CXCR4 pathway on aggrecanase-mediated cartilage loss.

Previous studies have shown a link between increased ADAMTS expression and matrix degradation, combined with a reduction in OA-associated cartilage loss in response to ADAMTS inhibition. Expanding on these initial findings, we provide in vitro evidence that CXCL12a promotes aggrecanase expression in primary chondrocytes (Figure 3). We also found that CXCL12a treatment induced greater RUNX-2 activation and less SOX-9 expression in primary chondrocytes pretreated with IL-1 (Figure 3), similar to the results of Kim et al. [21]. Their study revealed that CXCL12a promoted the proliferation and maturation of chondrocytes by increasing the expression of Runx2 and inducing the phosphorylation of p38 and Erk1/2 MAP kinases and IκB.

RUNX-2 is a major transcription factor regulating aggrecan loss via the regulation of ADAMTS-4/5 gene expression [29,30] while SOX-9 acts as a master regulator of aggrecanases at both the transcriptional and posttranscriptional levels [31,32,33]. This distinction may explain why some markers appeared to be stronger at the protein level than at the transcript level. We found that the CXCL12/CXCR4 axis induced aggrecanase activation via the regulation of both RUNX-2 and SOX-9 expression. We also found that it was involved in the expression of ACAN, indicating that the CXCL12/CXCR4 axis plays a dual role in controlling the levels of ECM components. When this equilibrium cannot be sustained, ECM degradation and cartilage degradation occur. CXCL12a had weaker effects on the expression of SOX-9 in IL-mediated primary chondrocytes, suggesting that this phase of cartilage development has a more limited capacity for repair and regeneration, often resulting in chondrocyte dysfunction. The effects on ADAMTS-5 appeared to be stronger at the protein level than at the transcriptional level, suggesting the involvement of posttranscriptional effects.

Three signaling pathways implicated in aggrecanase activity, including MAPK, NF-κB, and Wnt/β-catenin, were also examined in the context of CXCL12a. Numerous MAPKs have been shown to play a role in proteolytic cartilage degradation [34,35]. Inhibition of these pathways has been associated with decreased expression of RUNX-2 and ADAMTS-5 in cultured human chondrocytes, while activation of these pathways results in strong RUNX-2 expression and aggrecanase expression [36,37]. In contrast, the activation of MAPKs has also been shown to enhance the expression of SOX-9 in primary chondrocytes [38]. This sophisticated regulatory mechanism offers some clues regarding the differential expression of SOX-9. In addition to these pathways, NF-κB signaling is another regulator controlling aggrecanase expression, which also suppresses SOX-9 activation [39,40,41].

The Wnt/β-catenin pathway is also crucial in the pathogenesis of OA, affecting multiple cellular pathways in chondrocytes [42], including increased expression of proteinases and suppression of SOX-9 [43,44]. The activation of the MAPK, NF-κB, and canonical Wnt signaling pathways induced by CXCL12a described here provides additional proof of CXCL12a-medated aggrecanase activation, further establishing the role of the CXCL12/CXCR4 axis in aggrecanase activation and cartilage degradation (Figure 5c).

Taken together, our data strongly support the notion that PTOA-associated aggrecan loss can be prevented by blocking the CXCL12/CXCR4 pathway in vivo, further establishing the role of the CXCL12/CXCR4 axis in PTOA development. Our findings indicate that CXCL12/CXCR4-mediated aggrecanase upregulationaggrecanaseupregulation occurs due to activation of the MAPK, NF-κB, and Wnt/β-catenin pathways, with significant interactions and overlaps seen among pathways. Further investigations are required to determine the specific role of each signaling pathway in CXCL12/CXCR4 axis-mediated aggrecanase activation.

## 4. Materials and Methods

### 4.1. Reagents and Antibodies

AMD3100 (ab120718) and primary antibodies for CXCL12 (ab25117), CXCR4 (ab124824), ACAN (ab36861), SOX-9 (ab3697), RUNX-2 (ab76957), ADAMTS-4/5 (ab185722, ab41037), the aggrecan neo-epitope 374ARGSV (ab3773), PNCA (ab29), SB202190 (ab120638), U-0126 (ab120241), and C59 (ab142216) were purchased from Abcam (Cambridge, UK). Antibodies for ERK (#9102), phospho-ERK (#4377), JNK (#9258), phospho-JNK (#4668), p38 (#8690), phospho-p38 (#4511), p65 (#8242), phospho-p65 (#3033), IκBα (#4812), phospho-IκBα (#2859), β-catenin (#9582), GSK-3β (#12456), SURVIVIN (#2808), Cyclin D1 (#2978), and MMP-7 (#3301) were purchased from Cell Signaling Technology (Beverly, MA, USA). CXCL12a and CXCL12b ELISA kits were purchased from Tsz Biosciences (Boston, MA, USA). Alzet osmotic mini-pumps (2004) were purchased from DURECT Corporation (Cupertino, CA, USA). Recombinant rat CXCL12a and other reagents were purchased from Beyotime (Shanghai, China).

### 4.2. Animals and Experimental Design

Sprague-Dawley rats were purchased from the Experimental Animal Center of Tongji Medical College (Wuhan, China). All animals were housed at the animal care facility of Tongji Medical College at 25 °C with a 12:12 h light:dark cycle with food and water ad libitum. Animal studies were approved by the Institutional Animal Research Committee of Tongji Medical College, with all animal experimental protocols approved by the Institutional Animal Care and Use Committee (Approved number: TY20130286; Date: 14 December 2013).

For OA induction, 28 8-week-old male Sprague-Dawley rats (200 g ± 10 g) were divided randomly into three groups. DMM/AMD3100-treated rats (*n* = 9) underwent destabilization of the medial meniscus (DMM) on the right knee, as described by Glasson et al. [45]. Rats in this group were treated with AMD3100 (3 mg/day) via a constant infusion osmotic mini-pump (model 2004). DMM/PBS-treated rats (*n* = 9) underwent the same microsurgery of the right knee as described for the first group, followed by treatment with phosphate buffered saline (PBS) via constant infusion osmotic mini-pump. The sham control group (*n* = 10) underwent sham surgery on the right knee and received empty pumps. All animals were euthanized eight weeks after surgery. Given the lifespan of the Alzet osmotic pump, the pumps were exchanged once.

### 4.3. Cell Culture and Treatment

Primary chondrocytes were obtained from the knee cartilage of newborn Sprague-Dawley rats. In brief, isolated bones were cut into pieces in sterilized PBS. Then they were incubated in trypsin-EDTA with moderate rocking for 20 min at 37 °C and digested with 3 mg/mL collagenase (Beyotime, Shanghai, China) in complete Dulbecco’s modified essential media (DMEM) for 2 h. After the fractioned cells were filtered and collected, they were cultured in 2:3 DMEM: F12 medium supplemented with 10% fetal bovine serum (FBS) and 0.25% l-glutamine. Primary chondrocytes at passage 0 were utilized for all experiments due to their capacity for dedifferentiation during repeated passages [46].

### 4.4. Western Blot

Total protein was extracted from cells and quantified using the BAC Protein Assay Kit (Thermo Scientific, Rockford, IL, USA). Proteins (20 μg per well) were resolved on 12% SDS-polyacrylamide gels and transferred onto PVDF membranes (Millipore, Boston, MA, USA). Transferred membranes were blocked in 5% fat free milk at room temperature for 1 h, and then incubated with primary antibodies, according to the manufacturer’s instructions. Then the membranes were washed three times in TBST, and incubated with correspondent secondary antibodies at room temperature for 1 h. Immunoreactive proteins were visualized using enhanced chemiluminescence substrate (BOSTER, Wuhan, China) and ChemiDoc™ XRS+ System with Image Lab™ Software (BIO-RAD, Hercules, CA, USA). β-Actin antibodies (Cell Signaling Technology, Boston, MA, USA) were used as a control.

### 4.5. Real-Time Reverse Transcription-Polymerase Chain Reaction Analysis

Quantitative real-time reverse transcription-PCR (qRT-PCR) was performed as described previously [47,48]. Briefly, total RNA was extracted from rat chondrocytes using an RNeasy mini kit (Invitrogen, Carlsbad, CA, USA) according to the manufacturer’s instructions. First-strand cDNA was synthesized using MMLV reverse transcriptase (Promega, Madison, WI, USA). Templates were amplified using the SYBR Green Master Mix (Invitrogen, Carlsbad, CA, USA). All reactions were performed four times, and target gene expression was standardized to GAPDH. All primers used for quantitative real-time RT-PCR are listed in Table 1.

### 4.6. Enzyme-Linked Immunosorbent Assay for Synovial Fluid

Synovial fluid was collected from healthy and PTOA rats before they were sacrificed. Concentrations of CXCL12a and CXCL12b in the synovial fluid were determined using rat CXCL12a and CXCL12b ELISA kits (TszBiosciences, Boston, MA, USA).

### 4.7. RNA Interference Assay

Small interfering RNA (siRNA) against CXCR4 was designed by Transheep Bio (Shanghai, China). The sequence of CXCR4 siRNA was as follows: 5′-GCGAGGUGGACAUUCAUCUTT-3′ (sense), 5′-UUCUCCGAACGUGUCACGUTT-3′ (antisense). Gene silencing was performed according to the manufacturer’s instructions. Briefly, primary chondrocytes were cultured in a six-well plate for 24 h. When cell density reached 30%–50%, 5 μL lipofectamine 2000 (Invitrogen, Carlsbad, CA, USA) was diluted in 250 mL Opti-MEM medium, and then incubated for 5 min at room temperature. At the same time, CXCR4 siRNA or control siRNA was diluted in 250 μL Opti-MEM medium. Lipofectamine 2000 was gently mixed with each siRNA suspension. All samples were incubated for 20 min at room temperature. The siRNA–lipid complex was added to samples and incubated at 37 °C for 6 h. Finally, Opti-MEM medium was replaced and cells were incubated in 2:3 DMEM:F12 medium supplemented with 10% FBS and 0.25% l-glutamine for 24 h before drug treatment.

### 4.8. Histological Assessment

After animals were euthanized, we isolated, fixed, decalcified, and embedded knee joints and stained frontal sections through the entirety of the joint with 0.02% safranin orange and hematoxylin. We histologically assessed the cartilage samples obtained from rat knees with the modified Mankin scoring system to evaluate the severity of osteoarthritic changes [49,50]. All samples were assessed by three blinded independent observers, with final scores determined using an average for each joint.

### 4.9. Immunohistochemistry and Immunofluorescence

For immunohistochemistry, we analyzed paraffin sections of rat cartilage samples using the Dako REAL™ EnVision™ Detection System (Dako, Glostrup, Demark), followed by counterstaining with or without 0.02% safranin orange. For immunofluorescence, cells or sections were fixed and subjected to primary antibodies, followed by a goat anti-rat-FITC IgG (Invitrogen, Camarillo, CA, USA) as a second antibody. DAPI staining was used to display the nuclei. Color intensity of staining was quantified in five high-power fields using Image Pro Plus (Media Cybernetics, Rockville, MD, USA).

### 4.10. Statistical Analysis

Experiments were performed at least three times with similar results. All data are given as means ± 95% confidence intervals. Student’s *t* test was used to compare differences between two groups; the ANOVA method was used to compare groups. Statistical significance was defined as *p* < 0.05.

## 5. Conclusions

Our data suggest that the CXCL12/CXCR4 axis plays a pivot role in aggrecanase activation and cartilage degradation. Inhibition of the CXCL12/CXCR4 signaling axis slows aggrecanase-mediated catabolic processes and lessens the pathological progress of osteoarthritis.

## Figures and Tables

**Table 1:** Oligonucleotides used for quantitative real-time RT-PCR.

Target Rat Gene	Sequence
GAPDH	(F) 5′-GGCACAGTCAAGGCTGAGAATG-3′
	(R) 5′-GGTGGTGAAGACGCCAGTA-3′
Aggrecan	(F) 5′-CTTCCCAACTATCCAGCCAT-3′
	(R) 5′-TCACACCGATAGATCCCAGA-3′
ADAMTS-4	(F) 5′-GGAATGGTGGAAAGTATTGTGAGG-3′
	(R) 5′-GGTCGGTTCGGTGGTTGTAG-3′
ADAMTS-5	(F) 5′-CTCCATGCAGCTTTCACTGT-3′
	(R) 5′-CAGAATTTGGAATCGTCGTG-3′
SOX-9	(F) 5′-ACATCAAGACGGAGCAAC-3′
	(R) 5′-TGAAGGTGGAGTAGAGCC-3′
RUNX-2	(F) 5′-CCGCACGACAACCGCACCAT-3′;
	(R) 5′-CGCTCCGGCCCACA AATCTC-3′

Forward (F) and reverse (R) primers are listed. GAPDH = glyceraldehyde-3-phosphate dehydrogenase; ADAMTS-4 = a disintegrin and metalloproteinase with a thrombospondin motif 4; ADAMTS-5 = a disintegrin and metalloproteinase with a thrombospondin motif 5; RUNX-2 = Runt-related transcription factor 2; SOX-9 = SRY-type HMG box 9.
